# Supplementary material for: Differential responses of lung and intestinal microbiota to SARS-CoV-2 infection: a comparative study of the Wuhan and Omicron strains in K18-hACE2 Tg mice
Source: Lab Anim Res. 2025 Apr 23;41:11. doi: 10.1186/s42826-025-00241-x (PMC12016229; doi:10.1186/s42826-025-00241-x)
Supplement: Supplementary file 1 — Supplementary Material 1 [file 42826_2025_241_MOESM1_ESM.docx]

**
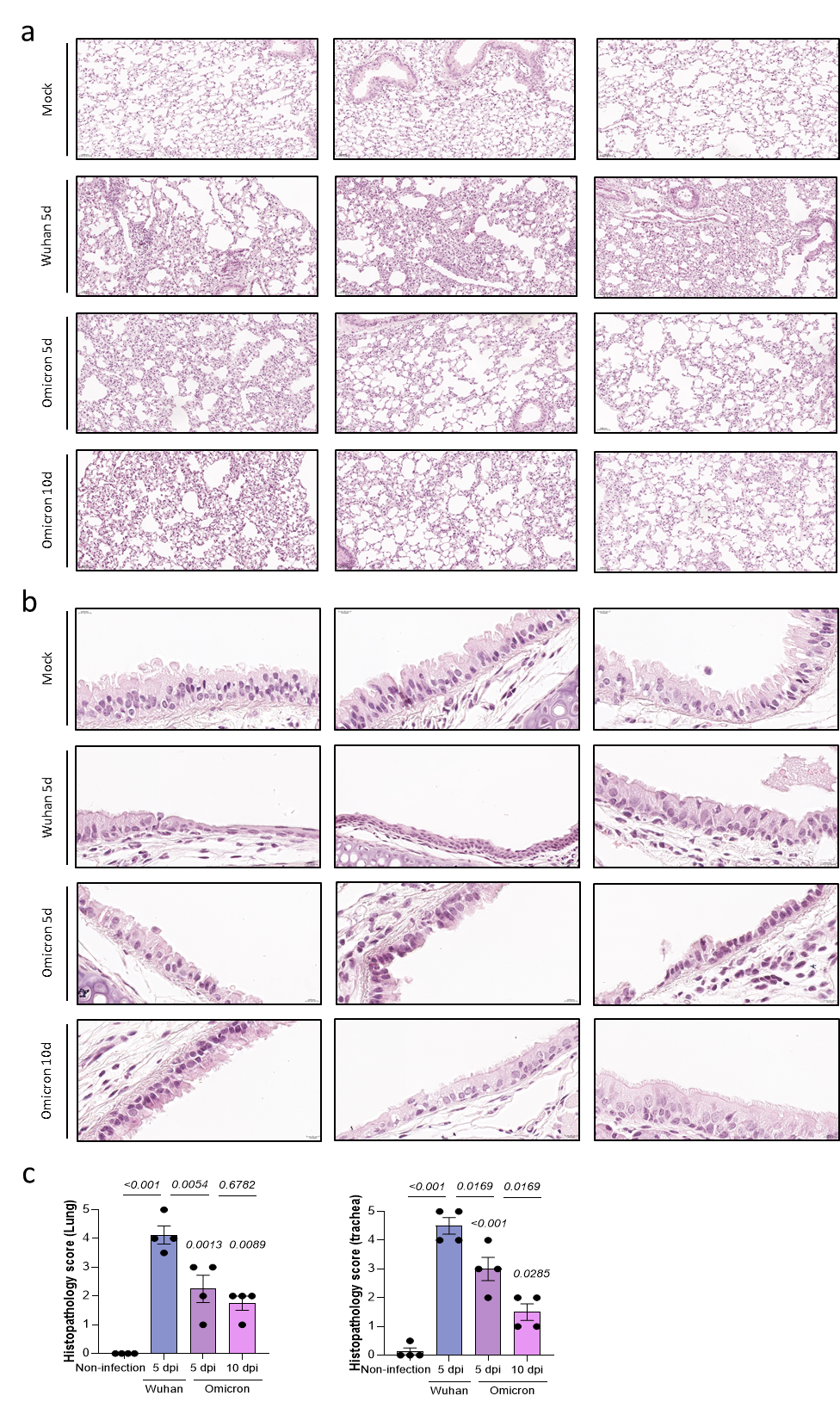
**

**Fig. S1.** **Histopathological analysis of the respiratory tract in non-infected, Wuhan, and Omicron-infected mice.**

(a-b) Histopathological analysis was performed using hematoxylin and eosin (H&E) staining. Non-infected mice (n = 4), Wuhan-infected mice (5 days post-infection [dpi], n = 4), and Omicron-infected mice (5 dpi and 10 dpi, n = 4 for each group) were analyzed in the lung (a) and trachea (b). (c) For the pathological score, three images per individual mouse were evaluated. The bar plots display the mean ± standard error of the mean (SEM), and the dots represent individual mouse images from each group. P-values were determined using one-way analysis of variance (ANOVA).
